# The Therapeutic Potential of Angeli’s Salt in Mitigating Acute *Trypanosoma cruzi* Infection in Mice

**DOI:** 10.3390/pathogens12081063

**Published:** 2023-08-19

**Authors:** Vera Lúcia Hideko Tatakihara, Aparecida Donizette Malvezi, Rito Santo Pereira, Bruno Fernando Cruz Lucchetti, Lucas Felipe Dos Santos, Rubens Cecchini, Lucy Megumi Yamauchi, Sueli Fumie Yamada-Ogatta, Katrina M. Miranda, Waldiceu A. Verri, Marli Cardoso Martins-Pinge, Phileno Pinge-Filho

**Affiliations:** 1 Laboratório de Imunopatologia Experimental, Departamento de Imunologia, Parasitologia e Patologia Geral, Centro de Ciências Biológicas, Universidade Estadual de Londrina, Londrina 86057-970, Paraná, Brazillucas.felipesantos@uel.br (L.F.D.S.); 2 Laboratório de Fisiologia e Fisiopatologia Cardiovascular, Departamento de Ciências Fisiológicas, Centro de Ciências Biológicas, Universidade Estadual de Londrina, Londrina 86057-970, Paraná, Brazil; 3 Departamento de Fisioterapia, Centro Universitário do Vale do Araguaia, Barra do Garças 78603-209, Mato Grosso, Brazil; 4 Laboratório de Fisiopatologia e Radicais Livres, Departamento de Imunologia, Parasitologia e Patologia Geral, Universidade Estadual de Londrina, Londrina 86057-970, Paraná, Brazil; 5 Laboratório de Biologia Molecular de Microrganismos, Departamento de Microbiologia, Centro de Ciências Biológicas, Universidade Estadual de Londrina, Londrina 86057-970, Paraná, Brazil; 6 Department of Chemistry and Biochemistry, University of Arizona, Tucson, AZ 85721, USA; 7 Laboratório de Pesquisa em Dor, Inflamação, Neuropatia e Câncer, Departamento de Imunologia, Parasitologia e Patologia Geral, Centro de Ciências Biológicas, Universidade Estadual de Londrina, Londrina 86057-970, Paraná, Brazil

**Keywords:** Chagas disease, therapy, nitroxyl, leukopenia, thrombocytopenia, oxidative stress, macrophages

## Abstract

Chagas disease (CD), caused by *Trypanosoma cruzi*, is a neglected tropical disease prevalent in Latin America. Infected patients are treated to eliminate the parasite, reduce the cardiomyopathy risk, and interrupt the disease transmission cycle. The World Health Organization recognizes benznidazole (BZ) and nifurtimox as effective drugs for CD treatment. In the chronic phase, both drugs have low cure rates and serious side effects. *T. cruzi* infection causes intense tissue inflammation that controls parasite proliferation and CD evolution. Compounds that liberate nitric oxide (NO) (NO donors) have been used as anti-*T. cruzi* therapeutics. Currently, there is no evidence that nitroxyl (HNO) affects *T. cruzi* infection outcomes. This study investigated the effects of the HNO donor Angeli’s salt (AS) on C57BL/6 mice infected with *T. cruzi* (Y strain, 5 × 10^3^ trypomastigotes, intraperitoneally). AS reduced the number of parasites in the bloodstream and heart nests and increased the protective antioxidant capacity of erythrocytes in infected animals, reducing disease severity. Furthermore, in vitro experiments showed that AS treatment reduced parasite uptake and trypomastigote release by macrophages. Taken together, these findings from the murine model and in vitro testing suggest that AS could be a promising therapy for CD.

## 1. Introduction

Chagas disease (CD) is a medical condition first identified by the Brazilian physician Carlos Chagas in 1909, and it arises from an infection with the protozoan *Trypanosoma cruzi*. CD continues to be a significant public health concern, particularly in endemic regions of Latin America, where it mainly spreads through contact between humans and the feces or urine of triatomine bugs that feed on blood. CD is endemic in 21 Latin American countries, with an estimated 6 to 7 million people currently infected. The World Health Organization (WHO) estimates that around 65–70 million people are at risk of infection [1]. Therefore, it is crucial to raise awareness about the scale of the problem, mobilize resources for prevention and treatment, and address the socioeconomic factors that perpetuate its transmission.

Due to the migration of infected individuals and non-vector transmission pathways such as blood transfusion, organ transplantation, and congenital infection, CD has emerged as a potential menace to non-endemic countries, particularly in the United States, Europe, and the Western Pacific region [2,3]. In areas endemic to CD, chronic cardiomyopathy associated with the condition is the leading cause of cardiac-related morbidity [4,5].

Although benznidazole (BZ) and nifurtimox have been utilized to treat CD since the 1960s [6], no satisfactory treatment is currently available for the chronic stage of the disease. These drugs have both limited efficacy and are often associated with side effects. Additionally, require long periods of administration, making it essential to develop new and effective alternatives to treat CD. Experimental infection of various animals with *T. cruzi* has facilitated the study of the natural history of CD. Research involving mice, rats, dogs, rabbits, guinea pigs, hamsters, and monkeys has yielded a wealth of information about the immunopathology underlying CD since its discovery [7,8]. Nonetheless, the major aspects of CD pathogenesis remain poorly understood. Over the past three decades, experimental investigations have demonstrated that BALB/c mice, when acutely infected with *T. cruzi*, produce interferon-gamma (IFN-γ) and tumor necrosis factor-alpha (TNF-α). This immune response leads to the activation of inducible nitric oxide synthase (iNOS) and an increase in the production of nitric oxide (NO), which is crucial for macrophage trypanocidal activity [9,10]. However, it is important to highlight that cytokines and NO also suppress host immunity [11].

Experimental findings also indicate that NO may play a role in regulating various other processes that are crucial for an effective immune response against the parasite [12,13]. The discovery that *T. cruzi* infection induces NO production within the myocardium has raised new inquiries, and the consequences of this discovery remain a topic of ongoing explorations [14,15].

**Figure 1:**
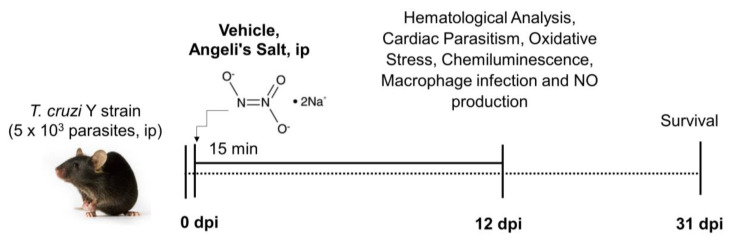
Experimental design. C57BL/6 mice were subjected to daily treatment with varying doses of Angeli’s salt (6, 60, and 600 µg/kg/animal), diluted in phosphate buffer (PBS) with a pH of 7.2 and administered via the intraperitoneal route. This treatment was administered 15 min post-infection for a duration of 12 days. During the acute phase, parasitemia was monitored by counting blood-borne trypomastigotes, while the assessment of survival was observed daily until day 31 post-infection. At 12 days post-infection, plasma nitrite level, oxidative stress, and macrophage infection were measured. All blood analyses and cell counts were conducted using standard methods.

Nitroxyl (HNO) arises from the protonation of NO following one-electron reduction [16], as shown in the Figure 1. It has been shown to act as a regulator of cardiovascular function and has been investigated as a potential treatment for heart failure, myocardial infarction, and hypertension, among other cardiovascular conditions [17] and hyperalgesia [18]. It functions as an endothelium-relaxing factor and hyperpolarizing factor [19]. However, it is a different entity than the related nitrogen oxides NO and NO^+^ [20,21]. Our investigation focused on examining the pharmacological properties of Angeli’s salt (AS; Na_2_N_2_O_3_), a known nitroxyl (HNO) donor [22], in *T. cruzi* infection pathogenesis.

## 2. Materials and Methods

### 2.1. Ethical Approval

The handling of animals and experimental procedures were carried out in strict adherence to the Guide for the Care and Use of Laboratory Animals, as recommended by the Brazilian National Council of Animal Experimentation (COBEA). The Internal Scientific Commission and the Ethics in Animal Experimentation Committee of Londrina State University approved the study protocol in compliance with the guidelines of the National Council for the Control of Animal Experimentation (CONCEA) under the Approval Number: CEUA no. 4628.2016.40/002.2021. All necessary measures were taken to reduce the pain and suffering of the animals involved in the experiments.

### 2.2. Animals and Experimental Design

We obtained male C57BL/6 mice (8–12 weeks old) from the Multidisciplinary Center for Biological Research (CEMIB), University of Campinas (UNICAMP), Campinas, Brazil. Subsequently, the mice were housed under standard conditions within the animal facility of the Department of Immunology, Parasitology, and General Pathology at the Center for Biological Sciences, State University of Londrina. 

The housing environment maintained a controlled ambient temperature of 21–23 °C and followed a 12-h light/dark cycle. The mice were provided with a commercial rodent diet (Nuvilab-CR1, Quimtia-Nuvital, Colombo, Paraná, Brazil) and had access to sterilized water ad libitum. For infection, we employed the Y strain of *T. cruzi* I lineage [23,24], which was generously provided by Dr. Paulo Maria Ferreira de Araújo from the Institute of Biosciences, Campinas State University, Campinas, São Paulo, Brazil.

The strain was maintained by weekly intraperitoneal (i.p.) inoculation of Swiss mice with 2 × 10^5^ trypomastigote forms present in the blood. To obtain infective blood trypomastigotes, blood was drawn via cardiac puncture from *T. cruzi*-infected mice under anesthesia. The motile blood forms were counted, and the desired number of parasites (5 × 10^3^) was then injected intraperitoneally (i.p.) into mice (n = 5–10 per group). Control groups received an injection of the same volume (100 µL) of 0.01 M sterile phosphate-buffered saline (PBS) (pH 7.2). For in vitro experiments, trypomastigotes grown and purified from a fibroblast cell line (LLC-MK2) were utilized.

To quantity parasitemia, 5 µL of fresh blood was collected from the tail vein of mice. The number of parasites within 50 microscopic fields at a 400× magnification was counted using an Olympus CH30LF100 (Olympus Optical CO., Ltd., Tokyo, Japan) light microscope. This counting procedure was performed on days 3, 5, 7, 9, 13, 15, 19, 21, 23, 25, 27, 29, and 31 post-infection. The data obtained were expressed as the number of parasites per mL [25]. Confirmation of *T. cruzi* infection was performed through direct microscopic observation of circulating trypomastigotes in the peripheral blood of mice at three days post-infection (dpi). The survival rate was assessed at 31 dpi (Figure 1). Only mice with positive parasitemia were included in the infected groups. To euthanize the animals, anesthesia was induced using ketamine (100 mg/kg) and xylazine (10 mg/kg) before performing cervical dislocation.

### 2.3. Treatment Schemes

The synthesis and utilization of AS (sodium trioxodinitrate) were executed in accordance with previously described methods [18,26]. Stock solutions were prepared in 10 mM NaOH and subsequently stored at −20 °C. The stability of solutions was determined based on their respective extinction coefficients at 250 nm (ε of 8000 M^−1^ cm^−1^ for AS [27] and prepared at 7 mg/mL in 10 mM NaOH). Mice were treated with PBS-diluted AS (at doses of 6 µg/kg/animal, 60 µg/kg/animal, and 600 µg/kg/animal), with these solutions being prepared daily, 15 min prior to the beginning of each treatment section. The treatment regimen was initiated 15 min after *T. cruzi* inoculation and continued for 12 consecutive days. Control groups, encompassing both infected and uninfected animals, were subjected to an equivalent volume (100 µL) of PBS (pH 7.2), as per the schedule for the intraperitoneally infected mice. The experimental design is depicted in Figure 1.

### 2.4. Hematological Analysis

Mice were anesthetized, and whole blood was collected through intracardiac puncture using syringes and needles containing ethylenediaminetetraacetic acid (EDTA) at a concentration of 2.0 mg per milliliter of blood. Standard methods were utilized to count the leukocytes, platelets, and reticulocytes [28,29]. Following this, plasma was separated and stored at −20 °C for subsequent use. The hematocrit values were obtained by microcentrifugation of capillary tubes filled with, while a manual hemocytometer was used to determine the total number of nucleated cells collected. The normal values obtained from uninfected C57BL/6 mice kept under the same conditions were considered in all experiments performed to determine the hematological parameters of the animals (12 dpi).

### 2.5. Cardiac Parasitism

At the 12th dpi, the hearts were collected and immersed in a 10% buffered formalin solution. The fixed tissues were embedded in paraffin, processed for microtomy, and subsequently stained with hematoxylin and eosin (H&E). Then, the tissue sections were examined under light microscopy, and the number of parasite nests within each section was counted across 50 microscope fields at a magnification of ×400. This evaluation was conducted on three distinct sections, and the results were expressed as the average of these three sections. Images were captured by a video camera adapted to a BX43 microscope (Olympus, Tokyo, Japan). The images were analyzed utilizing the ImageJ software, an open-source Java-based image processing tool (http://imagej.nih.gov/ij/; accessed on 20 June 2023).

### 2.6. Evaluation of Oxidative Stress and Antioxidant Capacity in Erythrocytes

Blood samples from the control and experimental groups (12 dpi) were used to deter-mine erythrocyte oxidative stress. After removing the plasma and white blood cells from whole blood, the remaining erythrocytes were washed three times with PBS. Following this, erythrocytes were resuspended within the same buffer (PBS) in a proportion of 1:99 (*v*/*v*) and were used immediately for conducting analyses. Oxygen uptake and induction time (Tind) (induced by 0.2 mM tert-butyl hydroperoxide, t-BHP) were measured using a Clark-type oxygen electrode at 37 °C [30,31]. Tind value is closely associated with the intracellular capacity of protective antioxidants, while oxygen uptake serves as an indirect indicator of the vulnerability of erythrocyte membranes to lipid peroxidation triggered by t-BHT.

### 2.7. Oxidative Processes Induced by Tert-Butyl Hydroperoxide in Erythrocytes

Mouse erythrocytes from the control and experimental groups were collected by centrifugation (800× *g*, 10 min) at 25 °C, followed by a triple washing procedure using PBS. When required, a 1% erythrocyte suspension in PBS was meticulously prepared. To initiate the chemiluminescence (CL) reaction, 20 µL of t-BHT (30 mM) was added to a final concentration of 0.6 mM within a 1 mL volume of erythrocyte suspension [32]. The outcomes of the chemiluminescence measurements were quantified in terms of relative light units (RLU) using a TD20/20 luminometer (Turner Biosystems, Sunnyvale, CA, USA). Moreover, the relative content of pre-existing lipid hydroperoxide within erythrocyte membranes was determined using the integrated area under the curve.

### 2.8. Evaluation of Nitric Oxide (NO) 

Nitrite levels in the plasma (12 dpi) were determined as an indirect measure of NO. For this purpose, the cadmium–copper system was employed, followed by the Griess reaction, as described previously [33]. Briefly, 60 µL aliquots were deproteinized by adding 50 µL of 75 mM ZnSO_4_ solution (Merck, Darmstadt, Germany), vortexed, and centrifuged at 11,200× *g* for 2 min at 25 °C. Then, 70 µL of 55 mM NaOH solution (Merck, Germany) was added to each supernatant, which was again vortexed and centrifuged at 11,200× *g* for 5 min at 25 °C. The final supernatants were diluted in glycine buffer solution (45 g/L; pH 9.7) (Merck, Germany) at a buffer-to-supernatant ratio 5:1.

For the cadmium–copper system, cadmium granules stored in a 100 mM H_2_SO_4_ solution (Merck, Germany) were rinsed three times in distilled sterile water and added to a 5 mM CuSO_4_ solution in a glycine–NaOH buffer (15 g/L; pH 9.7) (Merck, Germany) for 5 min to obtain copper-coated cadmium granules. Cadmium treatment was used to convert all nitrates into nitrites in the biological samples, providing a more accurate estimation of total NO in the original samples.

The glycine-buffered diluted supernatants were treated with activated granules (600–1000 mg, approximately 1–2 granules) and gently stirred for 10 min. Aliquots (200 µL) were transferred to microfuge tubes for nitrite determination. Griess reagent (200 µL) was added to each tube, consisting of Reagent I (50 mg of N-naphthyl ethylenediamine in 250 mL of distilled water) and Reagent II (5 g of sulfanilic acid in 500 mL of 3 M HCl) (Sigma–Aldrich, St. Louis, MO, USA). The tubes were incubated for 10 min at room temperature and centrifuged at 11,200× *g* for 2 min at 25 °C. The resulting supernatants were used for nitrite determination, and 100 µL of each sample was added to triplicate wells of a 96-well microplate. 

A calibration curve was prepared by diluting NaNO_2_ (Merck, Germany) in distilled sterile water to obtain concentrations ranging from 125 µM to 0 µM. Subsequent absorbance readings at a wavelength of 550 nm were undertaken using a LabSystems Multiskan EX microplate reader (Thermo Scientific, Waltham, MA, USA), and the results were ex-pressed in units of µM nitrite.

### 2.9. Cellular Metabolic Assay through Resazurin Reduction in Trypomastigotes

In this study, the effects of AS on trypomastigotes forms of *T. cruzi* (Y strain) was investigated, focusing on cell viability as determined by the resazurin assay [34]. Resazurin sodium salt, obtained from Sigma–Aldrich, St. Louis, MO, USA, was stored at 4 °C, protected from light exposure. A resazurin solution at 2 mM was prepared in DMEM (pH 7.4) and underwent filter sterilization prior to its use.

Experiments were carried out in 96-well microplates containing 1 × 10^6^ parasites per milliliter. The plates were incubated at 37 °C for 24 h, testing different concentrations of AS (3, 6, 60, and 600 μM). After the incubation period, the DMEM medium was removed.

Subsequently, DMEM (200 μL) containing resazurin (20 μM) was added to each well, and the plates were subjected to an additional incubation of 2 h, and subsequently, the fluorescence intensity was quantified using a Glomax^®^ fluorescence reader set to excitations at 520 nm and emission at 580 nm. Viable cells with active metabolism can reduce resazurin, a blue compound, to form resorufin, a pink molecule that emits light at 580 nm. Controls included the incubation of trypomastigotes alone in the presence of culture medium (DMEM and NaOH 10 mM, utilized as the vehicle for AS), and H_2_O_2_ 1 mM (positive control). The assays were performed in quadruplicate.

### 2.10. Inflammatory Peritoneal Macrophages Culture

Groups of C57BL/6 mice (five to ten individuals) were subjected to intraperitoneal administration of 2 mL of a 5% thioglycollate solution [35]. After four days, the resulting elicited cells from the peritoneal exudates were collected in cold PBS. The murine peritoneum was washed with 5 mL of ice-cold serum-free RPMI. Peritoneal cells were pooled and left to adhere to a complete medium formulation comprising RPMI, 2 mM glutamine, 1 mM sodium pyruvate, 40 µg/mL gentamicin, and 10 mM HEPES, for 24 h in 24-well plates at 2 × 10^6^ cells/well. Each pooled peritoneal cell suspension was plated in triplicate. The non-adherent cells were removed by washing, and the adherent cells were cultured in the complete medium. Macrophages were seeded onto 13 mm round glass coverslips and washed with warm PBS prior to the interaction assays. Additionally, macrophages were also seeded at a density of 2 × 10^5^ cells per well in 96-well plates.

### 2.11. Treatment of Macrophages and Invasion Assay

To examine the impact of AS on the host cell ability for parasite internalization, elicited peritoneal macrophages that had been washed beforehand were incubated with AS (15 µM, 30 µM, and 60 µM) in a 5% CO_2_ atmosphere at 37 °C for 1 h prior to the experiments. After removing the medium containing AS, macrophages were incubated with trypomastigotes at a ratio of five parasites per cell. This interaction took place within 24-well plates containing 13-mm diameter round glass coverslips. The mixture was allowed to re-act for 2 h at 37 °C in a 5% CO_2_ atmosphere. The cells were washed three times, fixed with Bouin’s fixative, stained with Giemsa stain (Merck, Darmstadt, Germany), and observed under a light microscope at 1000× magnification. To serve as a positive control for *T. cruzi* infection, some macrophages were incubated in medium alone. 

To calculate the internalization index, we multiplied the percentage of infected cells by the mean number of parasites per infected cell [36,37]. The internalization indices of all treated macrophages were normalized to those of un-treated macrophages. The experiments were repeated three times, and six independent experiments were conducted. Infected peritoneal macrophages that were untreated were included in all experiments as controls. Light microscopy was used for quantification, and 500 cells were randomly counted. 

### 2.12. Cytotoxicity Assay

Cell viability was determined by the MTT assay, which measures mitochondrial activity in living cells. The cells were incubated with MTT for 4 h at 37 °C, and the resulting formazan crystals were dissolved using dimethyl sulfoxide. Negative controls were also used. The plates were read using a Bio-Rad Microplate reader at a test wavelength of 570 nm and a reference wavelength of 630 nm.

### 2.13. Production of Nitric Oxide (NO) by Macrophages Treated with AS

The level of NO produced by macrophages after AS treatment was determined by measuring the accumulated nitrite level in the culture supernatant. After 48 h of treatment, the culture supernatants were collected and mixed with an equivalent volume of Griess reagent in 96-well culture plates, and incubated at room temperature for 10 min. Subsequently, absorbance was measured at 550 nm, and nitrite concentrations were calculated by referencing a standard curve generated using known concentrations of sodium nitrite.

### 2.14. Statistical Analysis 

The results are presented as the mean ± standard error of the mean (SEM) from three independent experiments, with 5–10 samples per group in each experiment. The Shapiro–Wilk test was performed to evaluate normality of the data, and after checking non-normality, non-parametric tests were used. The non-parametric Friedman ANOVA paired test was used to evaluate the evolution over day 3 to 31 after infection. Parasitemia levels were estimated by the area under the curve (AUC) during the acute *T. cruzi* infection. Survival curves were compared using a log-rank (Mantel–Cox) test. For variables exhibiting normal distribution, we performed parametric testing as two-way analysis of variance (ANOVA) followed by the Tukey post-test. GraphPad Prism software, version 8 (La Jolla, CA, USA) was used for all statistical evaluations. All analyses were two-tailed, and *p* values under 5% were considered significant (*p* ≤ 0.05).

## 3. Results and Discussion

### 3.1. Correlation between T. cruzi Infection and AS Therapy Response in Mice

**Figure 2:**
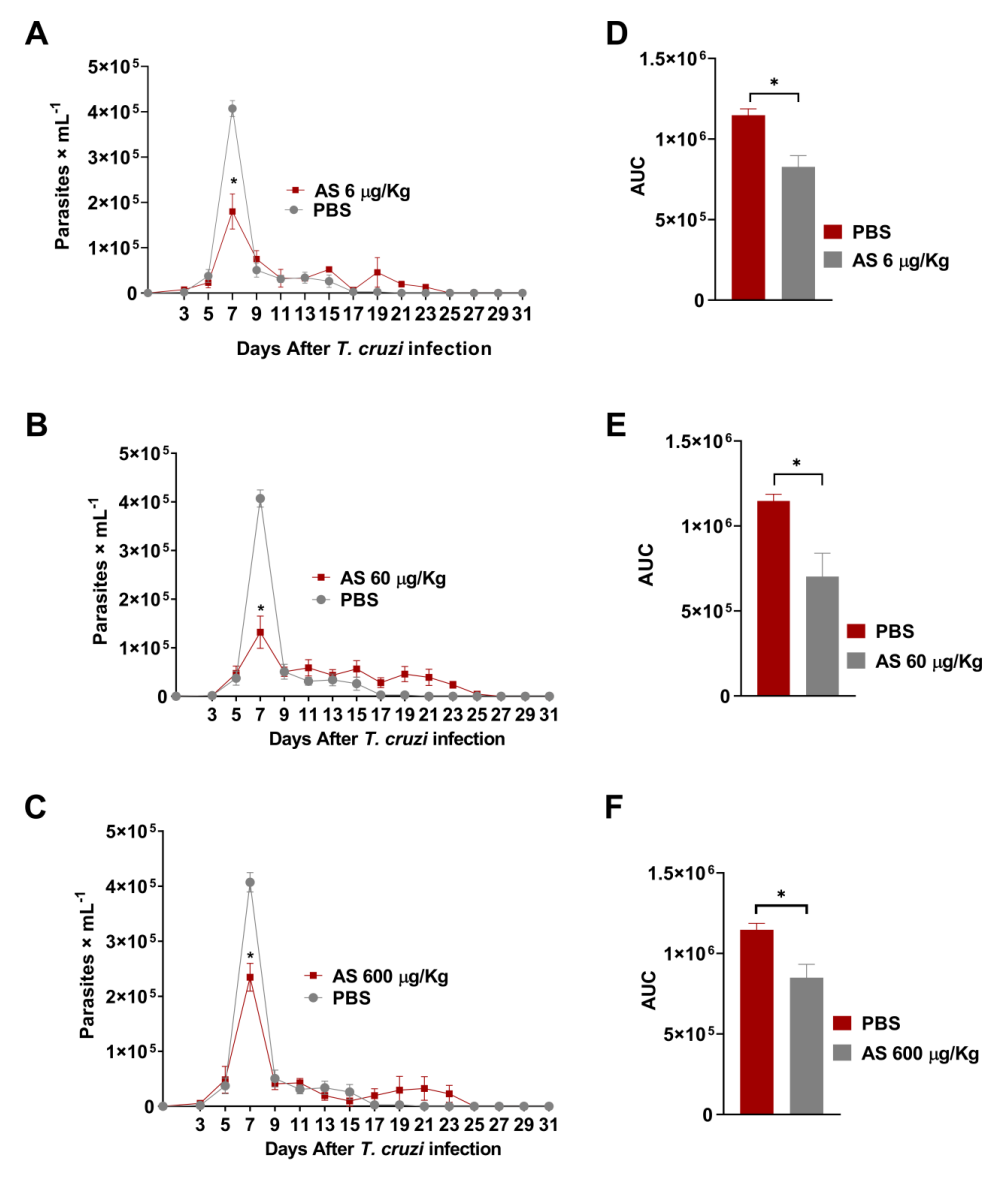
*T. cruzi* infection course and response to AS therapy. C57BL/6 mice were infected with 5 × 10^3^ trypomastigotes of *T. cruzi*. Daily treatment with AS (6–600 µg/kg/mouse) was initiated 15 min after infection and continued for 12 days. Control *T. cruzi*-infected mice received PBS (n = 8). The mean ± SEM values shown are representative of three independent experiments, and significant differences in parasitemia were observed (* *p* = 0.0067, Friedman test). (**A**) treatment with AS at doses of 6 ug/kg (n = 6), (**B**) 60 ug/kg (n = 8), and (**C**) 600 ug/kg (n = 7). Overall, parasitemia was also represented as the area under the curve (AUC) analysis. Significance was determined as * *p* = 0.0025 (**D**), * *p* = 0.00190 (**E**), and * *p* = 0.0091 (**F**), applying the unpaired *t* test.

NO is an important agent involved in *T. cruzi*-induced CD parasitemia and pathogenesis control [15]. The use of NO-based therapies to combat *T. cruzi* is a promising option, as demonstrated in various studies involving NO donor compounds, which have demonstrated the capacity to modulate NO levels both in vitro and in vivo [38,39]. However, the literature lacks comprehensive reports elucidating the impact of HNO on *T. cruzi* infections. In this study, we examined the anti-inflammatory and antiparasitic effects of AS, an HNO donor, in the context of acute *T. cruzi* infection. We found that AS improved resistance to *T. cruzi* infection both in vivo and in vitro. Figure 2 shows the parasitemia curves of *T. cruzi*-infected mice subjected to treatment with AS (AS group) and those receiving no treatment (PBS group). Six days post-inoculation (dpi) with *T. cruzi*, the parasitemia levels were similar between the two groups, with statistical analysis indicating no significant distinction (*p* > 0.05). The peak parasitemia level among AS-treated mice occurred at 7 dpi and was lower in comparison to the corresponding level in untreated mice (Figure 2A–C; *p* = 0.0067, Friedman test). This observation underscores the effect of AS therapy in the early phase of infection. Further evaluation of parasitemia were estimated by the area under the curve (AUC) during *T. cruzi* acute infection. Notably, the AUC observed in untreated infected mice was significantly higher than that observed in treated infected mice (Figure 2D, *p* = 0.0025; Figure 2E, *p* = 0.00190; Figure 2F, *p* = 0.0091, unpaired *t*-test). All the animals within the PBS group survived until 31 dpi. 

**Figure 3:**
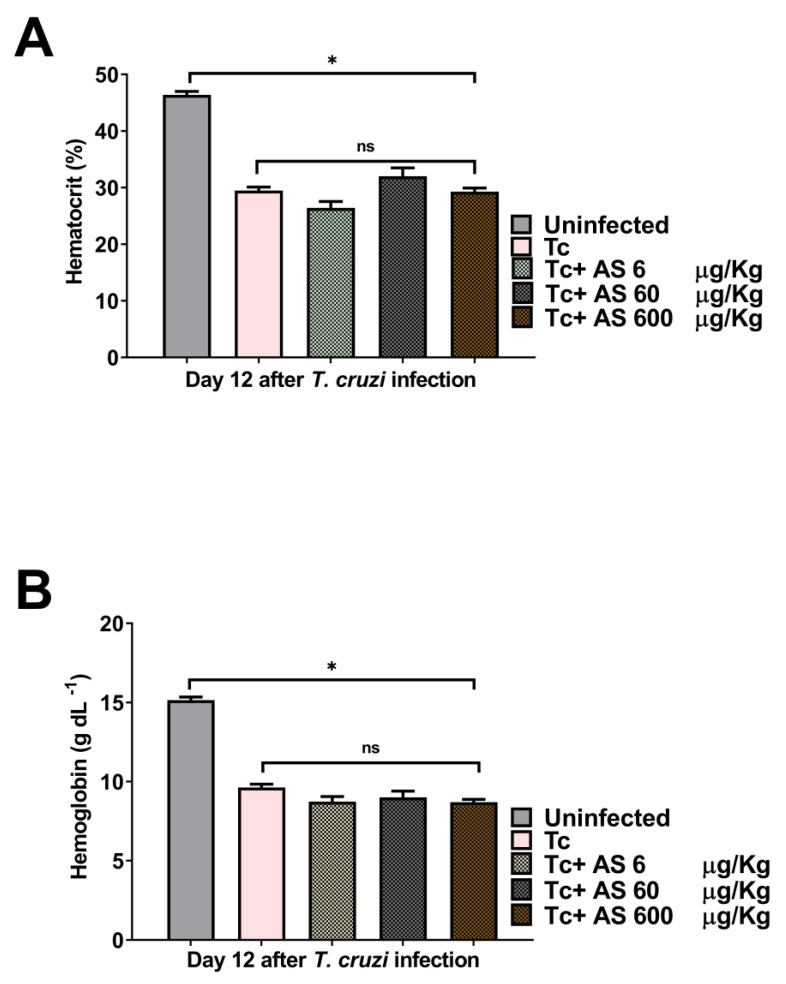
The effect of AS on anemia in *T. cruzi*-infected mice. On the 12th day post-infection, (**A**) hematocrit and (**B**) hemoglobin were evaluated. C57BL/6 mice were divided into groups of five and infected with 5 × 10^3^ *T. cruzi*, treated or not with AS (6–600 µg/kg/mouse). Control *T. cruzi*-infected mice (Tc group) received PBS. The mean ± SEM values shown are representative of three independent experiments, and significant differences were observed between uninfected and infected groups. * *p* ≤ 0.05, two-way ANOVA with Tukey post-test, ns = non significance.

Acute *T. cruzi* infection is associated with significant alterations in hematological parameters, such as anemia, thrombocytopenia, and leukopenia [28]. NO elevates circulating reticulocytes and reduces circulating leukocytes and neutrophils [30]. On the 12th dpi, we assessed blood cell counts and hematological values for both uninfected and *T. cruzi*-infected mice. Notably, we observed a significant decrease in hematocrit levels (Figure 3A) (*p* ≤ 0.05) and hemoglobin concentrations (Figure 3B) (*p* ≤ 0.05) in *T. cruzi*-infected mice, indicating the presence of anemia, as expected. AS treatment did not change these hematological parameters in infected animals (Figure 3A,B) (*p* > 0.05). 

**Figure 4:**
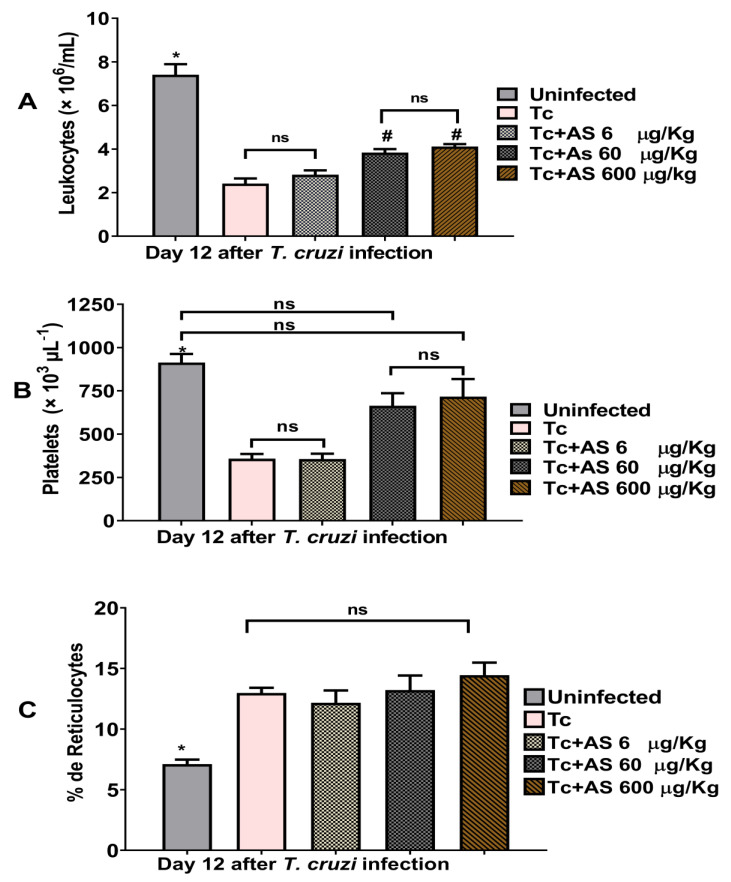
AS treatment mitigates leukopenia and thrombocytopenia observed during the acute phase of *T. cruzi* infection (day 12 p.i). For this investigation, C57BL/6 mice were divided into groups of five and infected with 5 × 10^3^ *T. cruzi*, treated or not with AS (6–600 µg/kg/mouse). The control group consisted of *T. cruzi*-infected mice (Tc group) receiving PBS. The mean ± SEM values shown in the figures are representative of three independent experiments. (**A**) Leukocytes, * *p* ≤ 0.05, two-way ANOVA with Tukey post-test (* uninfected vs. experimental groups), (# infected groups treated with AS 60 and 600 µg/kg/mouse group vs. uninfected group and vs. infected group treated with AS 6 µg/kg/mouse group). (**B**) Platelet levels and (**C**) reticulocyte counts, * *p* ≤ 0.05, two-way ANOVA with Tukey post-test (* uninfected vs. experimental groups), ns = non significance.

Unexpectedly, AS treatment exerted an impact on the degree of leukopenia and thrombocytopenia observed at 12 days post-infection (Figure 4A,B) (*p* ≤ 0.05), but did not alter the significantly higher number of reticulocytes in the *T. cruzi*-infected mice compared to the uninfected mice (Figure 4C) (*p* > 0.05). The importance of addressing thrombocytopenia and leukopenia in CD lies in their potential impact on an individual’s health. Thrombocytopenia can lead to excessive bleeding, while leukopenia can weaken the immune system, making individuals more susceptible to infections. These outcomes arise from the direct effects of the *T. cruzi* parasite on the bone marrow, where blood cells are produced [28]. Therefore, our data show that AS treatment left anemia and the increase in reticulocytes unaffected, while mitigating thrombocytopenia and leukopenia, both of which manifested on the 12th dpi during the acute phase of infection [30]. Experiments in our laboratory indicated that the treatment of BALB/c mice (a susceptible lineage for the Y strain) behaves differently after *T. cruzi*-infection; they experience “mild anemia” but have “high mortality rate” compared to the C57BL/6 mice used in this study [40]. Further studies will be necessary to determine the level of protection of AS in these more susceptible animals during the acute phase of the disease.

**Figure 5:**
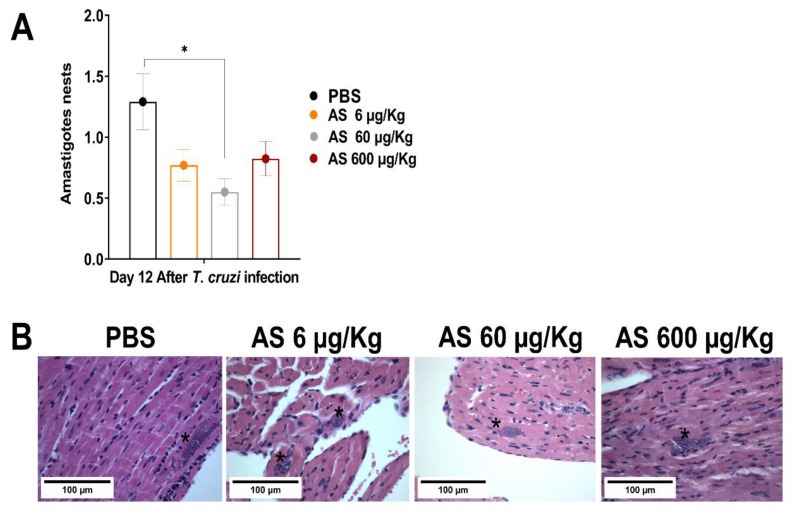
Effect of AS on heart parasitism. C57BL/6 mice were divided into groups of three to five individuals, and they were infected with 5 × 10^3^ *T. cruzi*, treated or not with AS (6–600 µg/kg/mouse). Control *T. cruzi*-infected mice received PBS. Animals were euthanized 12 days after infection, and sections of the heart from each mouse were collected for histopathological analysis. Tissue fragments were fixed in a 10% buffered formalin solution, dehydrated, cleared, and embedded in paraffin. Chilly tissue samples were sliced into 5 mm thick sections and stained with hematoxylin and eosin (H&E) to assess amastigote nests. (**A**) Tissue parasitism was scored by counting the total number of amastigote nests in 25 microscope fields (1 × 400 magnification) per histopathological section. The mean ± SEM values shown are representative of two independent experiments, * *p* ≤ 0.05, two-way ANOVA with Tukey post-test (* control vs. experimental groups). (**B**) Representative photomicrograph of cardiac tissue from control and experimental groups. * Shows amastigote nest.

AS treatment (60 µg/kg/mouse) markedly reduced the number of amastigote nests. Meanwhile, treatments with 6 µg and 600 µg AS showed a tendency to reduce the number of nests, but they were not statistically different from the infected and untreated animals (Figure 5A) (*p* > 0.05). In another context, mice treated with the NO-donating agents ruthenium nitrosyls (Ru(NO)isn or Ru(NO)imN) showed a reduced blood parasite load [38], and could eliminate amastigote nests in the myocardium tissue of infected BALB/c mice compared to the controls [39]. Thus, *T. cruzi*-infected mice treated with AS displayed promising results since the compound concentration (60 µg/kg/mouse) administered in the short-term treatment could significantly reduce the parasite load in the heart (Figure 5A,B) (*p* ≤ 0.05). However, it is pertinent to acknowledge that our study focused on examining the effects of AS on the course of *T. cruzi* infection and its impact on cardiac parasitism during the acute phase. Nevertheless, we did not investigate the progression of cardiac CD. Hence, further experiments are required to address and elucidate these aspects.

### 3.2. AS Attenuates Oxidative Stress in Erythrocytes 

**Figure 6:**
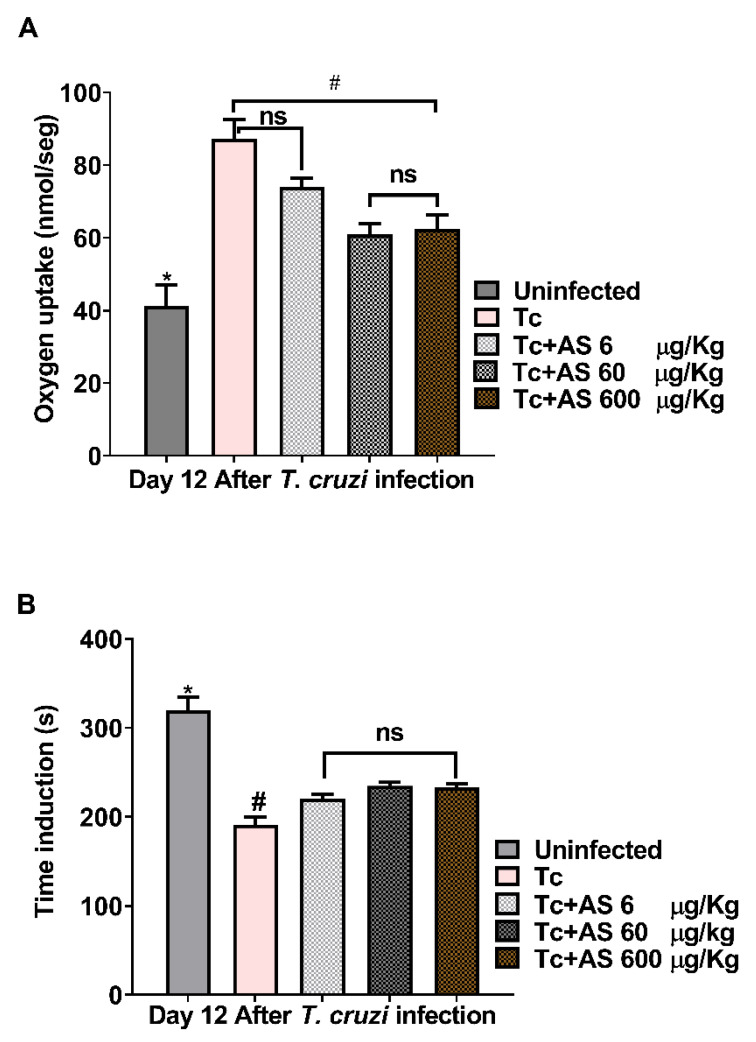
AS attenuates erythrocyte oxidative stress on day 12 after *T. cruzi* infection. (**A**) Oxygen uptake and (**B**) induction time. Groups of C57BL/6 mice (n = 5/group) were infected with 5 × 10^3^ *T. cruzi* and subsequently treated or not with AS (6–600 µg/kg/mouse). As a control, *T. cruzi*-infected mice (Tc group) received PBS. The mean ± SEM values shown are representative of two independent experiments, * *p* ≤ 0.05, two-way ANOVA with Tukey post-test (* uninfected vs. experimental groups), # *p* ≤ 0.05, two-way ANOVA with Tukey post-test (* control vs. experimental groups). ns = not significant.

The imbalance between antioxidants and oxidants is intricately linked to the progression of CD [41]. By the 12th dpi, erythrocytes from infected mice displayed a substantial increase in oxygen uptake (Figure 6A). Oxygen uptake in erythrocytes exposed to t-butyl hydroperoxide (t-BHP) exhibited an induction period, denominated Tind. An induction period refers to a delay or lag phase before a specific response or reaction occurs. In this case, it refers to a time interval during which the erythrocytes exposed to t-BHP do not immediately exhibit an increase in oxygen uptake. Remarkably, AS treatment reduced oxygen uptake at concentrations of 60 µg and 600 µg (Figure 6A) (*p* ≤ 0.05). Therefore, Tind is directly related to the intracellular protective antioxidant capacity of erythrocytes [31]. *T. cruzi* infection on the 12th day led to a significant reduction in Tind (Figure 6B) (*p* ≥ 0.05). AS treatment notably modified Tind (*p* ≤ 0.05) (Figure 6B) and improved the protective antioxidant capacity of erythrocytes within infected animals. In addition, we determined the temporal course of t-BHT chemiluminescence in erythrocytes, and our findings revealed an increase in chemiluminescence that correlated with oxidative stress previously induced in vivo. This led to the consumption of antioxidants and the formation of lipoperoxides, resulting in the emission of photons [32]. 

**Figure 7:**
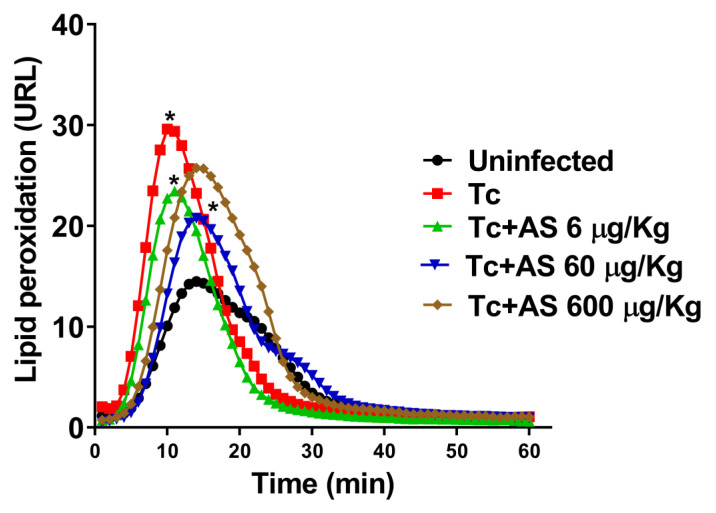
Time course curve of t-butyl hydroperoxide-initiated chemiluminescence in erythrocytes. Groups of C57BL/6 mice (n = 5/group) were infected with 5 × 10^3^ *T. cruzi* and either treated or not treated with AS (6–600 µg/kg/mouse). Uninfected mice and untreated *T. cruzi*-infected mice (Tc group) were included as controls. The values presented are the mean ± SEM and represent two independent experiments. Significance was determined as * *p* ≤ 0.05, using Kruskal–Wallis’s test, indicating a significant difference from the values observed in the controls (uninfected/infected-non-treated group or infected/non-treated group/infected treated group).

According to Figure 7, the profiles of mice infected with *T. cruzi* (Tc group) had significantly higher levels of lipoperoxidation compared to the controls (uninfected group) (*p* ≤ 0.05). Conversely, the AS-treated group demonstrated a significantly shorter initial rate of lipoperoxidation in contrast to the controls (Tc group), as indicated by the ascending segment of the curve in Figure 7 (*p* ≤ 0.05). Although the mechanism by which HNO impacts these parameters remains unknown, the data suggest that HNO donors hold therapeutic potential for preventing erythrocyte damage during the acute phase of CD.

### 3.3. AS Modifies Nitrite Levels in the Plasma 

**Figure 8:**
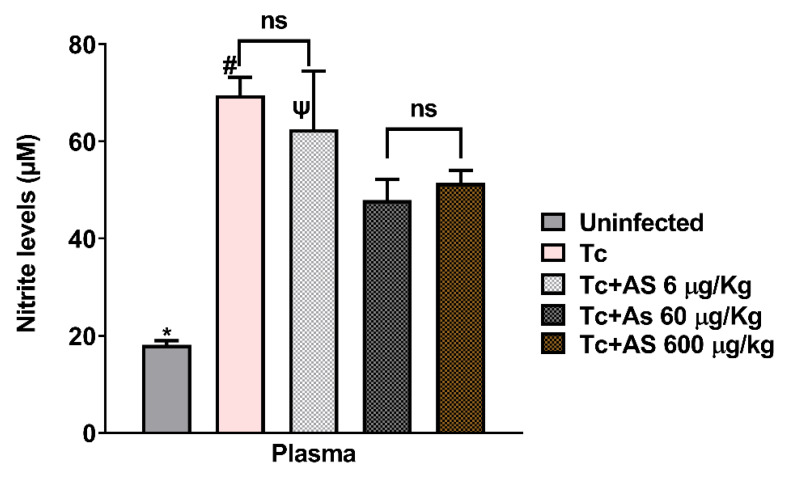
Effect of AS therapy on nitrite levels in the plasma (day 12 p.i). C57BL/6 mice were divided into groups and infected with 5 × 10^3^ *T. cruzi*, treated or not with AS (6–600 µg/kg/mouse). *T. cruzi*-infected mice (Tc group, n = 10) and uninfected mice received PBS (n = 9) and were used as controls. The mean ± SEM values shown are representative of two independent experiments, * *p* ≤ 0.05, two-way ANOVA with Tukey post-test (* uninfected vs. experimental groups), # *p* ≤ 0.05, two-way ANOVA with Tukey post-test (Tc vs. uninfected group and Tc vs. Tc 60 µg/kg), Ψ *p* ≤ 0.05, two-way ANOVA with Tukey post-test (Tc + 6 µg/kg vs. Tc 60 µg/kg and Tc + 6 µg/kg vs. Tc 600 µg/kg). Tc + 6 µg/kg (n = 5); Tc 60 µg/kg (n = 7); and Tc 600 µg/kg (n = 9). ns = non-significant.

As illustrated in Figure 8, nitrite levels in the plasma on day 12 of infection increased when compared to uninfected mice (18.11 ± 0.87 vs. 69.52 ± 3.68) (*p* ≤ 0.05), consistent with an anti-*T. cruzi* immune response. Interestingly, AS treatment reduced nitrite levels at the highest concentrations used: 60 µg/kg/mouse (69.52 ± 3.68 vs. 47.91 ± 4.29) (*p* ≤ 0.05) and 600 µg/kg/mouse (69.52 ± 3.68 vs. 51.42 ± 2.63) (p ≤ 0.05) (Figure 8). Lower nitrite levels, especially at the 60 mg/kg AS concentration used, are consistent with a reduced immune response and an increase in parasite counts, as shown in Figure 2B (after 11 days of infection).

### 3.4. AS Does Not Alter the Metabolic Activity of Trypomastigotes

**Figure 9:**
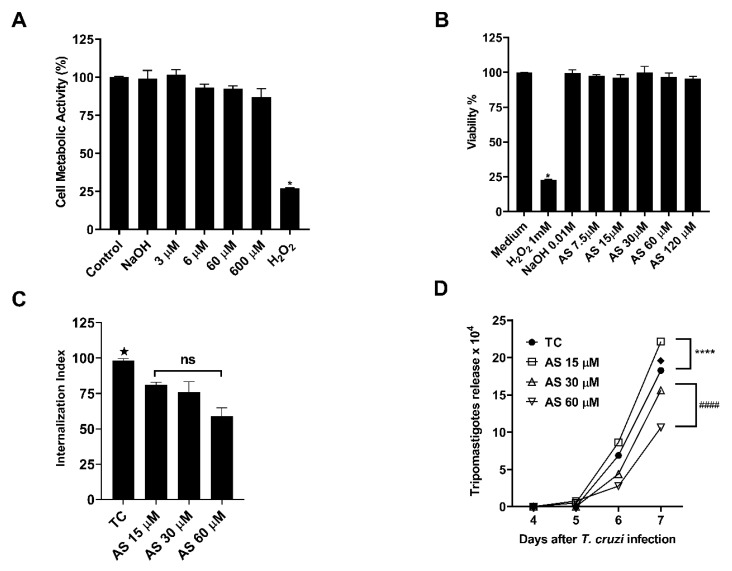
AS mediates *T. cruzi* infection in macrophages. (**A**) Cellular metabolic activity by reducing resazurin in *T. cruzi* trypomastigotes. (**B**) Cell viability in macrophages treated with AS (7.5–120 µM) by MTT assay. Controls consisting of H_2_O_2_ (1 mM) and NaOH (0.01 mM). (**C**) Internalization index of the interaction process between macrophages, treated with AS (15–60 µM) for 1 h and exposed to *T. cruzi* (5:1). (**D**) The effect of AS on trypomastigote release in *T. cruzi*-infected macrophages. Cells were infected with *T. cruzi* trypomastigotes and treated daily or not with AS. The release of trypomastigotes into the supernatant was detected and measured from day 4 to day 7 after infection. Values represent the mean ± SEM for triplicate determination and are representative of two independent experiments. * *p* ≤ 0.05, two-way ANOVA with Tukey post-test (H_2_O_2_ vs. experimental groups), ★ *p* ≤ 0.05, two-way ANOVA with Tukey post-test (Tc vs. experimental groups), ♦ *p* ≤ 0.0001, two-way ANOVA with Tukey post-test (Tc vs. experimental groups), **** *p* ≤ 0.0001, two-way ANOVA with Tukey post-test (Tc vs. AS 15µM), #### *p* ≤ 0.0001, two-way ANOVA with Tukey post-test (AS 30 µM vs. AS 60 µM). ns = non-significant.

AS concentrations ranging from 3 to 600 μM were evaluated for potential parasite toxicity, as shown in Figure 9A. Additionally, to validate the non-effectiveness of the AS antiparasitic activity against *T. cruzi*, parasites treated with AS and exhibiting 100% cellular metabolic activity were cultivated in a LLCMK-2 culture. 

### 3.5. AS Modulates T. cruzi Infection in Macrophages

The MTT assay was utilized to assess drug cytotoxicity in macrophages. AS treatment did not induce cell death (Figure 9B). To establish the effect of AS on the process of parasite invasion, peritoneal macrophages were treated with varying AS concentrations for 1 h before the macrophage invasion assay. To ensure that the treatment only affected host cells and not the parasites, the medium containing the NO donor was subsequently removed. Following a 2 h incubation with the trypomastigotes, the parasites were removed, and in some cases, AS was added to the medium every 24 h until the end of the *T. cruzi* infection period (7 dpi). Our findings show that AS treatment significantly decreased trypomastigote internalization by macrophages at 15 µM, 30 µM, and 60 µM concentrations (Figure 9C) (*p* ≤ 0.05). Additionally, AS reduced trypomastigote release into the culture supernatants from *T. cruzi*-infected macrophages, only at concentrations of 30 µM and 60 µM (Figure 9D) (*p* ≤ 0.0001). 

**Figure 10:**
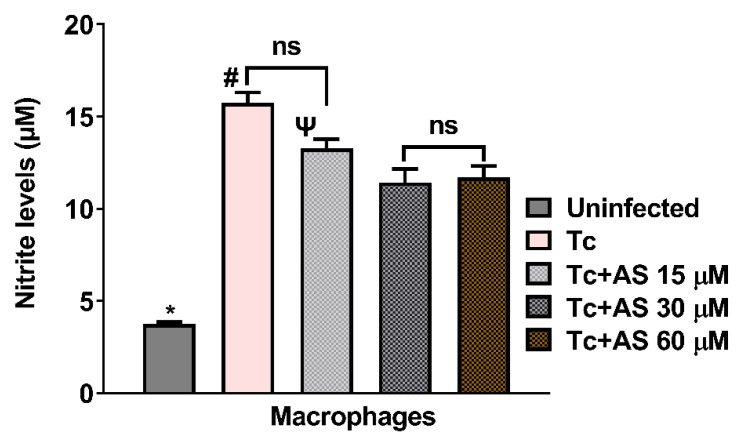
Effect of AS on nitric oxide (NO) production. Production of NO by macrophages was determined by measuring the level of accumulated nitrite, a metabolite of NO, in the culture supernatant using Griess reagent. Values are the mean ± SEM and are representative of two independent experiments. * *p* ≤ 0.05, two-way ANOVA with Tukey post-test (* uninfected vs. experimental groups), # *p* ≤ 0.05, two-way ANOVA with Tukey post-test (control vs. experimental groups), Ψ *p* ≤ 0.05, two-way ANOVA with Tukey post-test (Tc + 15µM vs. Tc 30 µM and Tc + 15 µM vs. Tc 60 µM). ns = non-significant.

We also investigated whether the production of NO by macrophages was influenced by treatment with AS. As shown in Figure 10, the nitrite levels in infected macrophages were elevated (*p* < 0.05), and AS treatment led to a decrease in nitrite levels in comparison with macrophages infected but not treated (*p* < 0.05). This reduction was also observed in the plasma of infected mice treated with AS at 12 dpi. The lower nitrite levels are consistent with a reduced immune response due to the higher number of parasites observed in the treated animals, compared to controls, from day 11 post-infection, as shown in Figure 2. Taken together, our data indicate that AS inhibits NO production, an important cytotoxic effector against *T. cruzi*. One hypothesis is that AS at the concentrations of 15, 30 and 60 µM of AS (Figure 9C), the available NO after treatment could effectively reduce the entry of trypomastigote forms. However, over time, NO concentration became insufficient to control the proliferation of trypomastigote forms (Figure 2). After 11 days of infection, the immune system may be struggling to control the parasite, and AS, by modulating the response to oxidative stress, may interfere with this immune response (e.g., modulating cytokine production such as IL-10 and IFN-gamma), resulting in increased difficulty in controlling the infection. Further studies focused on this aspect will provide better clarification.

## 4. Conclusions

Our research has revealed for the first time that AS targets *T. cruzi*, resulting in a decrease in parasite burden, accompanied by reduced levels of peroxidation of erythrocyte membranes and mitigation of thrombocytopenia and leukopenia during the acute infection phase. Additionally, AS induces alterations in the immune responses of infected macrophages, leading to enhanced anti-*T. cruzi* activity. The precise nature of these events is still unknown and may depend on the balance between host-produced NO and AS-released HNO, as well as the contribution of immunological mediators, such as pro or anti-inflammatory cytokines. Additional research is needed to thoroughly investigate the role of Angeli’s salt in the progression of cardiac CD, as well as its impact when administered at different time intervals (hours, days, weeks). Moreover, it is important to explore the potential effects of Angeli’s salt during the chronic phase of the disease in future studies. Promising strategies for the treatment of CD may involve the utilization of HNO donors in conjunction with BZ to mitigate the detrimental effects of oxidative damage in CD patients.

## Data Availability

Not applicable.
